# Pickering emulsion-templated polymers: insights into the relationship between surfactant and interconnecting pores

**DOI:** 10.1039/c9ra03186c

**Published:** 2019-06-17

**Authors:** Wenxiao Zhu, Yun Zhu, Ce Zhou, Shengmiao Zhang

**Affiliations:** Shanghai Key Laboratory of Advanced Polymeric Materials, School of Materials Science and Engineering, East China University of Science and Technology Shanghai 200237 China shmzhang@ecust.edu.cn

## Abstract

Pickering high internal phase emulsions (HIPEs) using micron-size polymeric particles as stabilizer were developed. By adding a small amount of surfactant to the Pickering HIPEs, macroporous polymers with a well-define open-cell structure were synthesized with these HIPEs as templates. Owing to the micron-size of the particles, the particle locations could be observed directly by laser scanning confocal microscopy. It was found that the excess and attached particles aggregated and formed thick particle layers around the droplets when the HIPE was stabilized solely by particles. These thick particle layers were extremely stable, and did not easily rupture during or after polymerization, which caused the resulting polymers to have a closed-cell structure. When a small amount of surfactant was added, it was found that the surfactant disaggregated the particles, leaving them well-dispersed in the continuous phase. Moreover, the surfactant tended to occupy the oil–water interface at the contact point of adjacent droplets, where the interconnecting pores were hence likely to be formed after consolidation of the continuous phase. This observation confirmed experimentally the mechanism of interconnecting pore formation in Pickering-HIPE-templated porous polymers proposed theoretically in previous works.

## Abstract 2

Pickering high internal phase emulsions (HIPEs) stabilized by micron-size polymer particles were developed, and the relationship between surfactant and interconnecting pores of these HIPE-templated porous polymers was experimentally clarified.

## Graphical abstract

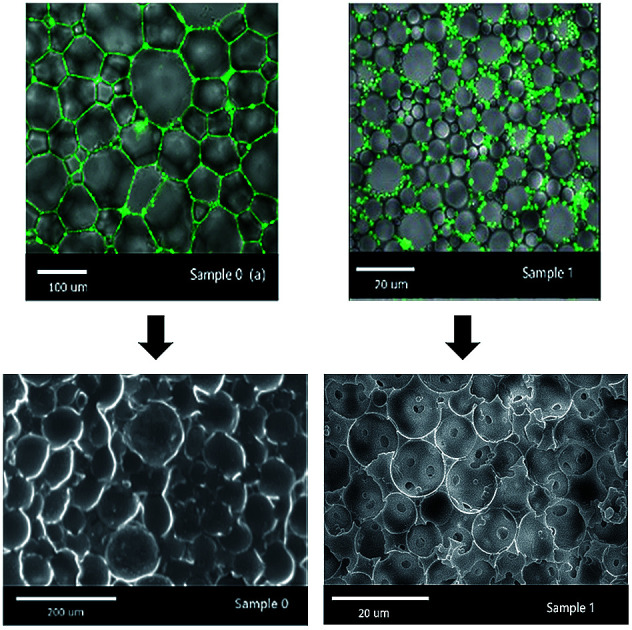

## 1. Introduction

Macroporous polymers have extensive applications in many fields, such as tissue engineering scaffolds,^1–4^ controlled release,^5–9^ ion adsorption,^10–13^ separation^14,15^ and supports for catalysis.^16–20^ These applications normally require porous polymers with an open-cell structure. One of the effective methods to synthesize macroporous polymers is the use high internal phase emulsion (HIPE) templates whose dispersed phase exceeds 74 vol%.^21–26^ If the continuous phase of HIPE contains polymerizable monomers, polymerization could be initiated to produce macroporous polymers (polyHIPEs).^27,28^ HIPEs are commonly stabilized by large amounts (5–50%)^27,29^ of non-ionic surfactants, which are difficult to remove completely after polymerization.^30^ One way to solve this problem is to substitute nanoparticles for surfactants, and these particle-stabilized HIPEs are commonly called Pickering HIPEs. There have been many nanoparticles reported for the stabilisation of HIPE, such as modified titania particles,^31,32^ silica particles,^33^ MOFs,^34–36^ and copolymer particles.^37–43^ In addition, the use of particles as stabilizers endows the resulting porous materials with a number of benefits. First, the particles used as stabilizers in Pickering HIPEs are irreversibly adsorbed at the water–oil interface because of their high energy of attachment, which makes the emulsion extremely stable. Second, the use of dispersed droplets in Pickering emulsions as templates can decorate the void walls of the resulting porous materials with a layer of solid particles, which could, for example, contain functional groups and lead to a variety of further applications.

However, it should be noted that the particle-stabilized HIPEs usually polymerize to form a closed-cell porous structure^44,45^ rather than an open-cell structure, as commonly observed in polyHIPEs from HIPEs stabilized with surfactant.^46^ Great efforts have been made to enhance the interconnectivity of porous polymers for the purpose of applications. There are two common approaches to enhance the interconnectivity and the permeability of the porous polymers. One is to control carefully the shrinkage of the continuous phase during the polymerization of HIPEs,^14,32,37,47–49^ However, this method is only applicable in certain HIPEs and is not versatile. The other is to add small amounts of surfactants into Pickering HIPEs before polymerization,^50–54^ which has been proved to be a more effective, convenient and universal method. Bismarck and his coworkers reported that well-defined open-cell porous polymers with silica particles stabilized Pickering HIPE by adding 5% Hypermer 2296 to the continuous phase.^50^ We also reported a series of open-cell porous polymers based on Pickering HIPEs by adding a small amount (∼1.0%) of surfactant to the emulsion before curing.^17,53–55^

The mechanism of formation of the closed-cell structure suggested that a thicker particle layer around the droplets formed very stable emulsion films which do not easily rupture during or after polymerization, so the closed-cell pore structure exhibits poly-Pickering-HIPEs.^56,57^ Furthermore, the formation of interconnected pores in the poly-Pickering-HIPEs following addition of a small amount of surfactant was assumed to proceed as follows. The surfactant adsorbs at the particle surface, leaving the particles well dispersed in the continuous phase and reducing the viscosity of the continuous phase. This results in thinner droplet films in comparison to the original Pickering-HIPE without surfactant.^50^ Moreover, the surfactant tends to occupy the oil–water interface at the contact point of adjacent droplets where no particles exist and the interconnecting pores are hence likely to be formed.^53^ Although, the mechanisms were clarified, direct experimental evidence of their existence in real emulsions is still missing. This may due to the fact that the size of the nanoparticles normally used to stabilize HIPEs in reported works is too small to be observed directly in the Pickering HIPEs.

**Scheme 1:**
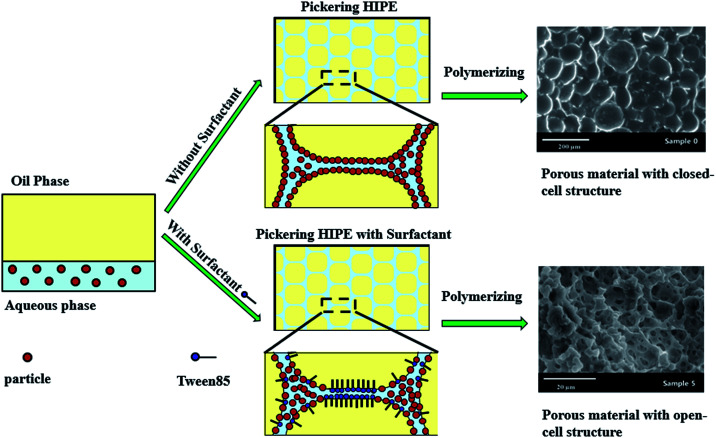
Preparation of polyHIPE based on Pickering HIPE with and without surfactant.

In this work, Pickering HIPEs stabilized by micron-size polymeric particles were prepared. The micron-size polymeric particles were prepared through dispersion copolymerization of styrene (St), divinylbenzene (DVB), and glycidyl methacrylate (GMA). Then, the polymeric particles were labelled with fluorescein isothiocyanate (FITC). Due to their micron-scale size and fluorescence labeling, the particles in these Pickering HIPEs were observed by laser scanning confocal microscopy (LSCM). Combined with SEM detection of the resulting poly-Pickering-HIPEs, it was found that a layer composed of closely packed particles surrounded the droplets in the HIPEs stabilized solely by particles, which hindered the formation of interconnected pores; and when a small amount of surfactant was added, it could be seen that disaggregated particles dispersed in the continuous phase and almost no particles were observed at the contact point of adjacent droplets (*i.e.* surfactant occupied the oil–water interface of the contact point of adjacent droplets), where the interconnected pores are hence likely to be formed. These observations provide direct experimental evidence for the formation of interconnected pores by addition of a small amount of surfactant (Scheme 1).

## 2. Experimental

### 2.1 Materials

Styrene (St, 99%, Shanghai Linfeng Chemical Reagent Ltd. Co.), divinylbenzene (DVB 80%, the remainder being *m*- and *p*-ethylstyrene, Aldrich) and glycidyl methacrylate (GMA 97%, Shanghai Macklin Biochemical Co., Ltd) were purified by passing through basic chromatographic aluminum oxide to eliminate the inhibitor before use. 2,2′-Azobis-(isobutyronitrile) (AIBN) (Adamas, Shanghai, China) was recrystallized from ethanol, then dried under vacuum at room temperature before use. Ethylenediamine (EDA), fluorescein isothiocyanate (FITC) and poly(vinylpolypyrrolidone) (PVP k-30) were obtained from J&K Chemical Ltd. Shanghai, China, and used as received. Acrylamide (AM), *N*,*N*′-methylenebis(acrylamide) (MBAM), and *N*,*N*,*N*′,*N*′-tetramethylethylenediamine (TMEDA) were purchased from Sigma-Aldrich and used as received. Paraffin (Admas, Shanghai, China) was used as received. Cyclohexane, and Tween85 were purchased from Sinopharm Chemical Reagent Co., Ltd., and used as received. Ammonium persulfate (APS, Lingfeng Chemical Reagents Co., Ltd. Shanghai, China) was recrystallized twice before use. Deionized water was used.

### 2.2 Methods

#### 2.2.1 Preparation of fluorescence labelled polymeric particles

Micron-size monodisperse poly(St–DVB–GMA) particles were synthesized by dispersion polymerization in an ethanol–water medium using AIBN as an initiator and poly(vinylpolypyrrolidone) (PVP k-30) as a stabilizer. A mixture of St (9.0 g), GMA (1.0 g), and AIBN (0.15 g) was added to an aqueous solution (90 ml) containing PVP of 1.0 g under stirring. Then this oil/water mixture was polymerized at 70 °C for 12 h.

The obtained polymer particles were separated from the mixture by centrifugation and washed with ethanol three times. Then, the polymer particles were dried to a constant weight and subsequently dispersed in 20 ml deionized water. Ethylenediamine (2.4 ml) was added to the polymer particle aqueous dispersion, and then the dispersion was left at 50 °C for 12 h in a nitrogen atmosphere. As a result, amine groups were introduced to the surface of these particles through the reaction with the epoxy group. With the amination finished, the aminated polymeric particles were washed with water and dried under vacuum. The dried polymeric particles were dispersed in a pH 9.0 sodium carbonate buffer with FITC, and magnetic stirring at room temperature for 24 h. Finally, FITC was introduced to label the polymeric particles.

#### 2.2.2 Preparation of poly-Pickering-HIPEs

Polymeric particles were initially dispersed into a 2.4 ml aqueous solution (see Table 1 and Scheme 1). Then, AM (0.6 g), MBAM (0.06 g), APS (0.02 g) and TMEDA (0.04 g) were dissolved in the above polymeric particle dispersion. The Pickering HIPE was prepared by mixing this aqueous solution (*i.e.* aqueous phase) with 10 ml of paraffin (*i.e.* an oil phase), and the mixture was homogenized by an Ultra Turrax T18 homogenizer (7.5 mm rotor) operating at 12 000 rpm for 2 min. The HIPEs thus obtained were then transferred to a sealed glass mold, and polymerized in a 40 °C oven for 24 h. The polymerized materials were removed from the molds, freeze-dried to a constant weight, then extracted in a Soxhlet apparatus with cyclohexane to remove any impurities. Finally, the resulting polymers were freeze-dried to a constant weight. In the case of Pickering HIPE stabilized by particles and surfactant, the surfactant was added to the aqueous phase before homogenization.

#### 2.2.3 Characterization of Pickering HIPEs and poly-Pickering-HIPEs

The particle size and distribution of the copolymer particles in the aqueous dispersion were determined by dynamic light scattering (DLS) with a Beckman Coulter LS230 particle size analyzer. The phase behavior of the Pickering HIPE was detected by tracking the delta backscattering of monochromatic light with *λ* = 880 nm from the emulsion using an optical analyzer (Turbiscan Lab Expert, Formulaction, France). As soon as the Pickering HIPE was prepared, it was moved to a flat bottomed cylindrical glass tube (the height and external diameter of this tube were 70 and 27.5 mm, respectively). Then, the glass tube was placed in the optical analyzer, and backscattering of light from the sample was periodically measured along the height at 25 °C. The results were presented as a sedimentation profile (backscattering *versus* time). The emulsion viscosity was characterized by a stress-controlled rheometer (HAAKE, MARS III) with parallel-plate geometry spaced 1 mm apart. The shear rate ranges from 0.01 to 40 s^−1^.

SEM images of Au-coated polymeric particles and porous materials were taken with a Hitachi S-3400N SEM. The average void diameter (*D*) of the poly-Pickering-HIPEs was estimated from the SEM images. At least 100 voids were measured for each sample. The average interconnecting pore diameter (*d*) was measured from the SEM images directly. At least 100 interconnecting pores were measured for each sample. The interconnectivity (*I*) of the polyHIPEs was calculated by *I* = *n* × (*d*/*D*)^2^, where *n* was the average number of interconnecting pores per void and was also calculated from SEM images. At least 50 voids were calculated for each sample. The optical microscopy images of Pickering HIPEs were taken on a Nikon laser scanning confocal microscopy.

## 3. Result and discussion

**Fig. 1:**
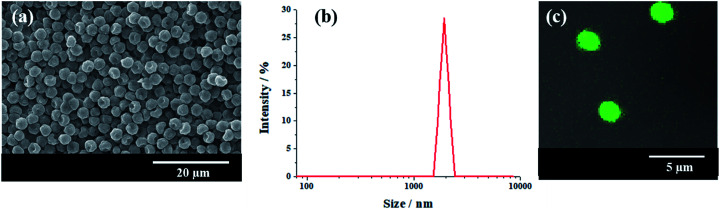
(a) SEM images of aminated particles, (b) DLS size distribution of aminated particles, and (c) LSCM images of FITC labeled particles.

In attempt to observe directly the particles in real Pickering HIPEs, micron-size polymeric particles were synthesized with a dispersion copolymerization of St, GMA and DVB, and this was followed by amination with ethylenediamine. The number-average size (*D*_*n*_) of the aminated polymeric particle is 1.9 μm, and PDI is 0.051 (Fig. 1). SEM observation showed that these particles were uniform (Fig. 1a and b). The dye FITC was introduced to label the polymeric particles successfully (Fig. 1c).

**Fig. 2:**
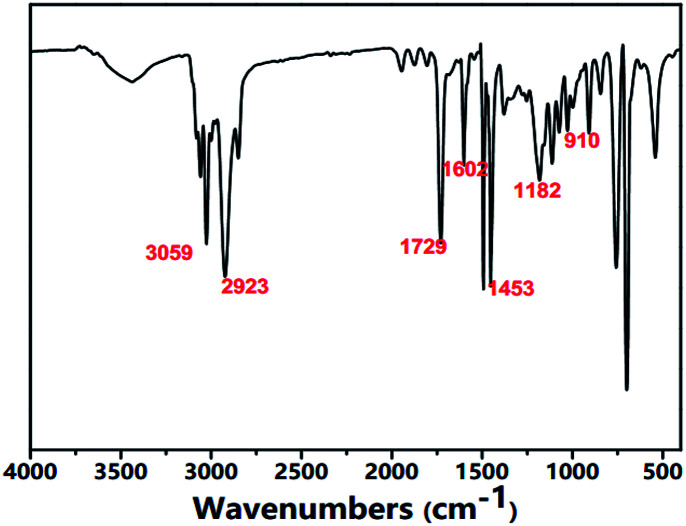
FT-IR spectra of aminated particles.

As shown in Fig. 2, the FTIR spectra of the aminated particles presented typical poly(St–DVB–GMA–NH_2_) particle structure characteristics. The peaks of 910 cm^−1^ and 849 cm^−1^ corresponded to the stretching vibrations of –CH(O)CH–; the bands of 1603 cm^−1^ were matched with the stretching vibrational absorption of the benzene ring. The typical absorption peak of –N–H could also be observed at 1573 cm^−1^. The peaks appearing at the wavenumbers of 2923 cm^−1^ and 1742 cm^−1^ arose from the ester group.

**Fig. 3:**
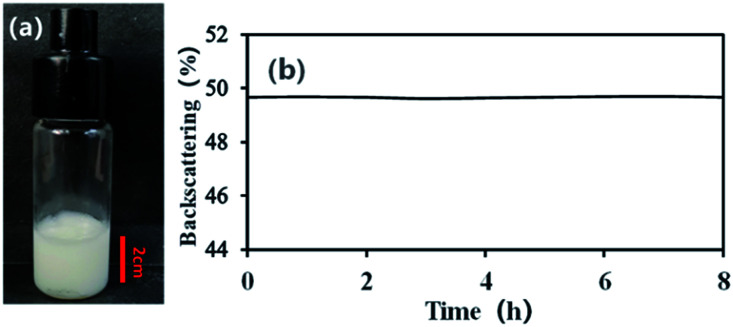
The O/W HIPE stabilized with polymeric particles of 10 wt% relative to the continuous phase. (a) Photograph of the HIPE placed for 8 h after prepared; (b) backscattering data of the HIPE within 8 h at 25 °C.

**Fig. 4:**
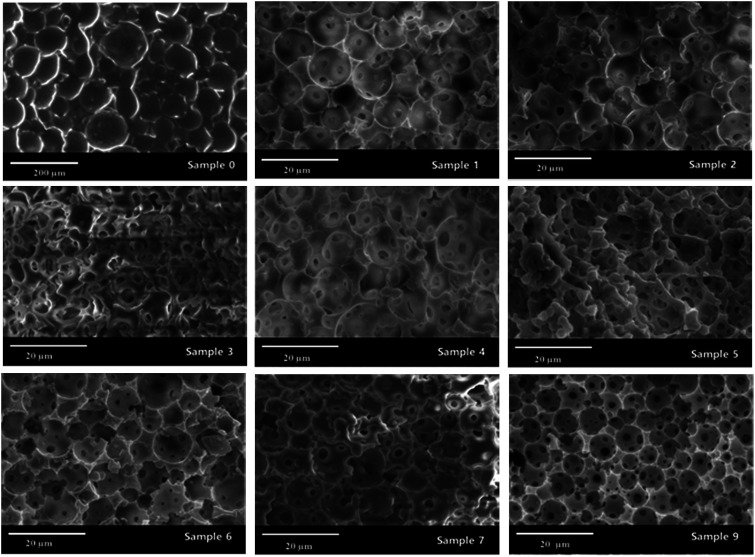
SEM images of porous materials. Sample 0 was the poly-Pickering-HIPE synthesized from a Pickering HIPE which was solely stabilized by 10 wt% polymeric particles. Sample 9 was the polyHIPE synthesized from a HIPE which was solely stabilized by 5.0 wt% Tween85. Samples 1–7 were the polyHIPEs synthesized from HIPEs which were stabilized by both polymeric particles and Tween85. The Tween85 concentration of samples 1–5 was 1.0 wt%, and the corresponding particle concentration: sample 1: 2.0 wt%; sample 2: 4.0 wt%; sample 3: 6.0 wt%; sample 4: 8.0 wt%; sample 5: 10 wt%. The Tween85 concentration of sample 6 and sample 7 was 2.0 wt%, and the corresponding particle concentration: sample 6: 2.0 wt%; sample 7: 4.0 wt%.

With an AM and MBAM aqueous solution containing polymeric particles of 10 wt% as the continuous phase, a paraffin-in-water (O/W) Pickering HIPE was obtained successfully (Fig. 3a). Turbiscan analysis showed that this HIPE was stable against coalescence for more than 8 h (Fig. 3b) which meant that the HIPE, stabilized solely by the micron-size polymeric particles, could be an ideal template to produce poly-Pickering-HIPE. As expected, the polymerization of this Pickering HIPE caused a closed-cell porous polymer (Fig. 4, sample 0) as reported in the literature.^44,45^ Moreover, the average void diameter of the poly-Pickering-HIPE (124 ± 8 μm; Fig. 4, sample 0) was much larger than that of the conventional polyHIPE (7.3 ± 1.0 μm; Fig. 4, sample 9) which was synthesized from O/W HIPE solely stabilized by Tween85 of 5 wt%, which agreed with the observation of poly-Pickering-HIPEs in previous works.^50,53^

**Fig. 5:**
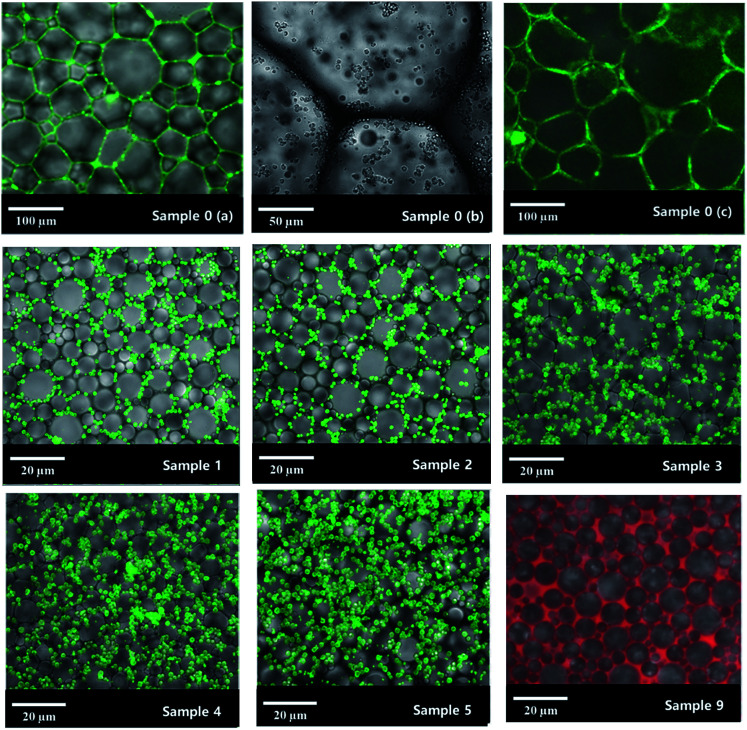
LSCM images of HIPEs. Sample 0 is the Pickering HIPE solely stabilized by 10 wt% polymeric particles: (a and b) are the Pickering HIPEs stabilized by fluorescent microspheres. Because the fluorescent intensity of the particles in the highly curved region was extremely strong, the particles at the boundary cannot be seen clearly. The non-fluorescent picture is shown in (b). (c) The polyHIPE whose corresponding HIPE is stabilized by fluorescent microspheres. Sample 9 was the HIPE solely stabilized by 5.0 wt% Tween85. whose continuous phase was dyed by Rhodamine B. Samples 1–5 were the HIPEs stabilized by both polymeric particles and Tween85 (at a concentration of 1.0 wt% in the aqueous phase). The corresponding particle concentrations: sample 1: 2.0 wt%; sample 2: 4.0 wt%; sample 3: 6.0 wt%; sample 4: 8.0 wt%; sample 5: 10 wt%.

**Fig. 6:**
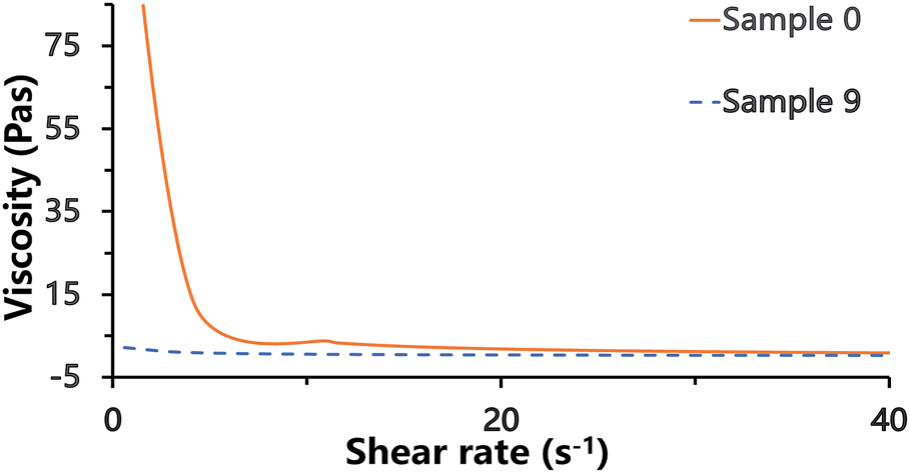
Viscosity of HIPEs. Sample 0 was the Pickering HIPE solely stabilized by 10 wt% polymeric particles; sample 9 was the HIPE solely stabilized by 5.0 wt% Tween85.

Laser scanning confocal microscopy (LSCM) observation of the Pickering HIPE showed that the droplets of this HIPE were surrounded by close-packed polymeric particles (Fig. 5, sample 0, Scheme 1), and increased the viscosity of the HIPE (compared to the HIPE stabilized solely by surfactant) (Fig. 6). This particle layer has been mentioned by Bismarck and his coworkers,^50^ and was extremely stable; it does not easily rupture during or after polymerization.^56,57^ Accordingly, the poly-Pickering-HIPE from the HIPE stabilized solely by polymeric particles is mainly closed-cell in form.

**Fig. 7:**
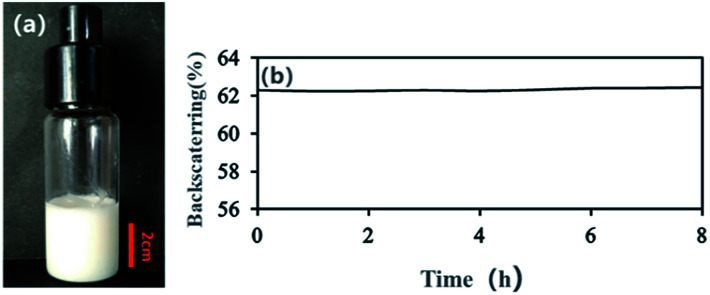
O/W HIPE stabilized with Tween85 and polymeric particles of 1.0 and 10 wt%, respectively. (a) Photograph of the HIPE 8 h after preparation; (b) backscattering data of the HIPE within 8 h at 25 °C.

To prevent the particles from aggregating (close-packing), the water soluble dispersant Tween85 of 1.0 wt% (respect to the aqueous phase) was added to the Pickering emulsion templates containing polymeric particles of 2–10 wt% (Table 1, samples 1–5), and the pore structure of its resulting poly-Pickering HIPE was investigated. Tween85 is a viscous but liquid non-ionic polymeric surfactant used as an effective dispersing agent and O/W emulsifier. In this work, HIPEs with Tween85 of 1, 3, or 5 wt% as the sole stabilizer were also prepared (Table 1, samples 8 and 9). However, the HIPEs with Tween85 less than 3 wt% were not stable enough to be templates for the preparation of porous polymers, because they underwent obvious separation during heating. This phenomenon meant that HIPE samples 1–5 prepared with addition of Tween85 of 1.0 wt% were stabilized by both polymeric particles and Tween85 (Fig. 7).

**Table 1:** Composition of emulsion templates and the parameters of the resulting polyHIPEs

Samples	Surf.^a^ (wt%)	Particle^a^ (wt%)	*D* b/μm	*d* c/μm	*I* d/μm
0	0	10	124 ± 6.2	0	0
1	1	2	12.4 ± 1.5	1.8	4.8
2	1	4	11.9 ± 1.0	1.9	6.6
3	1	6	10.1 ± 0.6	1.9	7.0
4	1	8	8.5 ± 1.6	1.4	10.7
5	1	10	8.2 ± 1.3	1.4	11.1
6	2	2	10.1 ± 1.2	1.5	5.2
7	2	4	9.8 ± 1.1	1.7	7.3
8^e^	3	0	—	—	—
9	5	0	7.3 ± 1.0	1.2	11.8

a With respect to the continuous phase.

b Average void diameters.

c Average interconnecting pore diameters.

d Interconnectivity, calculated by *n* × (*d*/*D*)^2^ × 100, where *n* is the average number of interconnecting pores per void.

e The preparation of polyHIPE based on HIPE sample 8 failed, because the HIPE separated when it was heated for an hour.

**Fig. 8:**
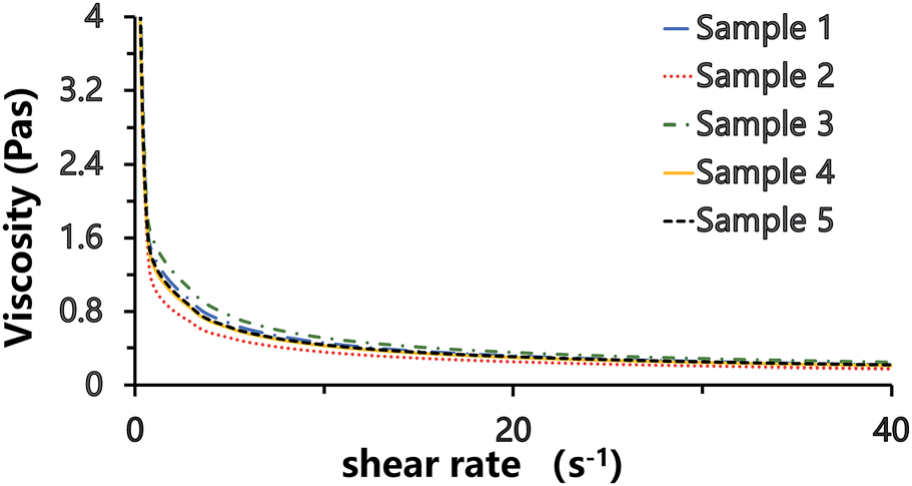
Viscosity of HIPEs. Samples 1–5 were the HIPEs stabilized by both polymeric particles and Tween85 (concentration of 1.0 wt% in the aqueous phase). The corresponding particle concentration: sample 1: 2.0 wt%; sample 2: 4.0 wt%; sample 3: 6.0 wt%; sample 4: 8.0 wt%; sample 5: 10 wt%.

The polymerization of this Pickering HIPE produced a series of poly-Pickering-HIPEs with a clear open-cell structure (Fig. 4, samples 1–5), which agreed with the morphology observed in the porous polymers based on the Pickering HIPEs stabilized by both nanoparticles and surfactants.^50,53^ To clarify the way in which the interconnecting pores in the void walls were formed, the HIPEs were observed by LSCM (Fig. 5). It was found that the micron-size polymeric particles were loosely distributed in an orderly manner on the water–oil interface of the droplets, and were absent from the contact area of the adjacent droplets when the particle content was low (*e.g.* 1 or 2 wt%, Fig. 5, samples 1 and 2). Furthermore, with an increase in particle content, such as 8 or 10 wt% (Table 1, samples 4 and 5), the particles distributed surrounding the droplets became irregular, and some excess particles remained in the aqueous phase. However, it was found that the particles in all the five samples were not aggregated (Fig. 5, samples 1–5), which was also confirmed by the viscosities of the HIPEs as detected by a rheometer. It was found that these five samples had a similar viscosity during detection (shearing rate from 0.01 to 40 s^−1^) (Fig. 8), which meant that there was no aggregation among the polymeric particles,^58,59^ because the surfactant disaggregated the particles. Moreover, vacancies (no polymeric particles) were observed in the contact area of the neighboring droplets, which meant that the surfactant adsorbed on the oil–water interface competitively with polymeric particles, and occupied the contact area of the neighboring droplets where the interconnecting pores were, hence, likely to be formed (Scheme 1). These observations provide direct experimental evidence for the formation of interconnected pores by the addition of a small amount of surfactant.

Although increasing the polymeric particle content did not hinder the formation of interconnecting pores (Table 1, samples 1–5), it decreased the average droplet size of the Pickering HIPEs (Fig. 5, samples 1–5) and consequently decreased the average void size of the resulting poly-Pickering-HIPEs (Fig. 4, samples 1–5). As shown in Table 1, when a Tween85 concentration of 1.0 wt% in the aqueous phase was used, varying the polymeric particles in the continuous phase from 2.0 to 10 wt% caused a steady decrease in the average void diameter from 12.4 to 8.2 μm. Furthermore, increasing the polymeric particle content from 2.0 to 10 wt% also increased the number of interconnecting pores in each void (Fig. 4, samples 1–5), and accordingly, the interconnectivity increased from 4.8% to 11.1% (Table 1). Hence, increasing the amount of polymeric particles used to stabilize Pickering HIPEs would enhance the HIPE stability to coalescence, which caused a smaller average void size and higher interconnectivity. This phenomena could be due to the fact that increasing particle content both provided more material to create a higher interface area and decreased the total void volume, thus creating smaller void volumes. This description was also confirmed by the SEM analysis of poly-Pickering-HIPEs from the Pickering HIPEs stabilized by varied particle content and Tween85 of 2 wt% (samples 6 and 7 in Table 1 and Fig. 4).

## 4. Conclusions

In this work, a Pickering HIPE stabilized by micron-size polymeric particles was prepared. Polymerizing this HIPE caused a closed-cell porous polymer. With addition of a small amount of the surfactant Tween85 to this Pickering HIPE, a series of pore-morphology-tunable porous polymers were obtained. Due to their micron-scale size and fluorescence labeling, the particles in the Pickering HIPEs could be observed with laser scanning confocal microscopy. Combined with SEM detection of the resulting porous polymers, it was found that a layer composed of closely packed particles surrounded the droplets in the HIPEs stabilized solely by particles, which hindered the formation of interconnected pores; and when a small amount of surfactant was added, it could be seen that disaggregated particles dispersed in the continuous phase (especially in the highly curved regions of the continuous phase), and almost no particles were observed at the contact point of adjacent droplets (*i.e.* surfactant occupied the oil–water interface of the contact point of adjacent droplets) where the interconnected pores are, hence, likely to be formed. These observations provide direct experimental evidence for the formation of interconnected pores in Pickering HIPE-templated polymers by the addition of a small amount of surfactant to the Pickering HIPEs.

## Conflicts of interest

There are no conflicts to declare.

## Supplementary Material
